# Circular RNA circ_0004470 accelerates the occurrence of lung cancer by promoting DNA damage and cell cycle arrest

**DOI:** 10.1016/j.jbc.2025.108456

**Published:** 2025-03-27

**Authors:** Xueqi Li, Yufei Liu, Qiaoxin Zheng, Weizhou Liu, Huanxuan Li, Shan Rao, Ziwei Xue, Qiuhan Hua, Meizhen Li, Yueting Shao, Xun Li, Yun Zhou, Yiguo Jiang

**Affiliations:** 1 The Key Laboratory of Advanced Interdisciplinary Studies, The First Affiliated Hospital of Guangzhou Medical University, Guangzhou, China; 2 Institute for Chemical Carcinogenesis, Guangzhou Medical University, Guangzhou, China

**Keywords:** lung cancer, CircRNA, DNA damage, BPDE, malignant transformation

## Abstract

Defects in the DNA damage response are associated with tumorigenesis, and circular RNAs (circRNAs) can also affect the occurrence and progression of cancer by regulating gene expression. However, the relationship between DNA damage in lung cancer and circRNAs remains unexplored. In this study, circ_0004470 was significantly upregulated in various lung cancer cells (H446, A549, H1299) as well as in carcinogenic animal models and clinical lung cancer samples. Circ_0004470 promoted DNA damage and cell cycle S phase arrest in human pulmonary bronchial epithelial cells, inhibited DNA repair, and accelerated malignant transformation in response to continuous DNA damage–inducing stimulation. Circ_0004470 inhibited DNA repair and cell cycle progression by binding specifically to the nucleotide excision repair complex Xeroderma pigmentosum group C-complementing protein and damage-specific DNA binding protein 1, thus interacting with the DNA damage response process and accelerating the accumulation of DNA damage. These findings suggest that circRNAs are involved in regulating genetic damage-associated lung cancer and provide insight into the mechanism by which circ_0004470 affects the DNA damage response during carcinogenesis.

Lung cancer persists as a major global health burden, with mounting epidemiological evidence implicating industrial pollutants as key etiological factors (1, 2). Data from the Global Burden of Disease Study 2019 indicate that polycyclic aromatic hydrocarbons (PAHs), solid fuels, and particulate matter pollution significantly increase the burden of disease for cancers of the trachea, bronchus, and lung (3, 4). Benzo[a]pyrene-7,8-diol-9,10-epoxide (BPDE), a reactive metabolite of benzo[a]pyrene, primarily forms bulky DNA adducts (*e.g.*, N^2^-dG-BPDE) by covalently binding to guanine residues, distorting DNA structure, and interfering with replication/repair. Although adducts are the initial lesions, secondary damage (*e.g.*, strand breaks, mismatches) can emerge during error-prone repair or replication stress: nucleotide excision repair (NER) generates transient single-strand breaks during adduct removal, whereas unresolved lesions may progress to double-strand breaks or crosslinks. Damage outcomes are context-dependent and influenced by exposure dose, duration, and cellular repair efficiency (5, 6, 7, 8). Despite well-characterized genotoxic effects, the molecular cascades linking chronic BPDE exposure to lung tumorigenesis remain incompletely defined.

Accumulation of unrepaired DNA lesions constitutes a fundamental oncogenic mechanism, facilitating proto-oncogene activation and tumor suppressor loss (9). The NER pathway serves as the primary defense against BPDE-induced bulky adducts. Mechanistically, Xeroderma pigmentosum group C-complementing protein (XPC)-HR23B complexes initiate lesion recognition in global genome repair (10). The clinical relevance of NER deficiency is supported by studies demonstrating that XPC-KO mice exhibit 2.0-fold increased susceptibility to CS-carcinogen–driven lung adenocarcinomas compared with WT controls (11). Environmental estrogens may impair DNA repair capacity by suppressing the transcriptional levels of XPC and other NER-related genes through promoter hypermethylation (12). This dual assault (direct DNA damage + repair pathway suppression) creates a permissive microenvironment for carcinogen-driven malignancy.

Emerging paradigms reveal that environmental carcinogens subvert genomic integrity through coordinated interference with DNA damage response (DDR) networks. Beyond inducing mutagenic lesions, these agents dysregulate critical cell cycle checkpoints. DNA binding protein 1 (DDB1) exemplifies this dual functionality: it facilitates NER through chromatin remodeling and concurrently regulates G1/S transition by targeting replication licensing factor CDT1 for proteasomal degradation (13). Exposure to exogenous DNA-damaging agents induces the DDB1–Cul4A ubiquitin ligase complex to specifically degrade cell cycle checkpoint proteins such as CDT1, thereby maintaining genomic integrity. Conversely, silencing DDB1 expression leads to the accumulation of unresolved DNA lesions, triggering checkpoint activation and genomic instability (14). Similarly, acetaldehyde promotes esophageal carcinogenesis not only *via* DNA crosslinking but also through ATR-Chk1 checkpoint inactivation, highlighting the multidimensional nature of environmental carcinogen action (15).

Recent advances implicate circular RNAs (circRNAs) as critical epigenetic regulators bridging carcinogen exposure and DDR outcomes. These covalently closed RNAs exhibit remarkable stability and functional versatility, operating through miRNA sponging, protein scaffolding, or direct transcriptional regulation. In hydroquinone-induced leukemia and malignant transformed cells, hsa_circ_0001944 interacts with the poly ADP ribose polymerase 1 (a key enzyme involved in DNA repair and transcription regulation) and human antigen R complex to regulate apoptosis, thereby affecting the progression of leukemia and malignant transformed cells (16). Our prior investigations established a mechanism of pollutant-specific circRNA regulation: nicotine-derived nitrosamine ketone (NNK) elevates circNIPBL to suppress base excision repair efficiency by 45%, whereas carbon nanoparticles induce circ_0089282 to recruit LIG4 and enhance NHEJ-mediated repair (17, 18). In this study, we established a BPDE-induced chronic exposure lung cancer model and identified significant upregulation of circ_0004470 promoting DNA damage accumulation, cell proliferation, and metastasis. Mechanistically, circ_0004470 amplified BPDE carcinogenicity by impairing XPC–DDB1 complex functionality, simultaneously compromising NER efficiency and cell cycle checkpoint control.

In this study, we elucidated the crosstalk between environmental genotoxins and epigenetic regulators in lung carcinogenesis. We demonstrated the dual role of circ_0004470's in impairing DNA repair and checkpoint enforcement, thereby providing a mechanistic framework for understanding how chronic pollutant exposure converts transient DNA damage into persistent oncogenic stress. These insights advance biomarker development and targeted therapeutic strategies against environmentally driven malignancies.

## Results

### DNA damage and cell cycle arrest persist during BPDE-induced lung carcinogenesis

**Figure 1:**
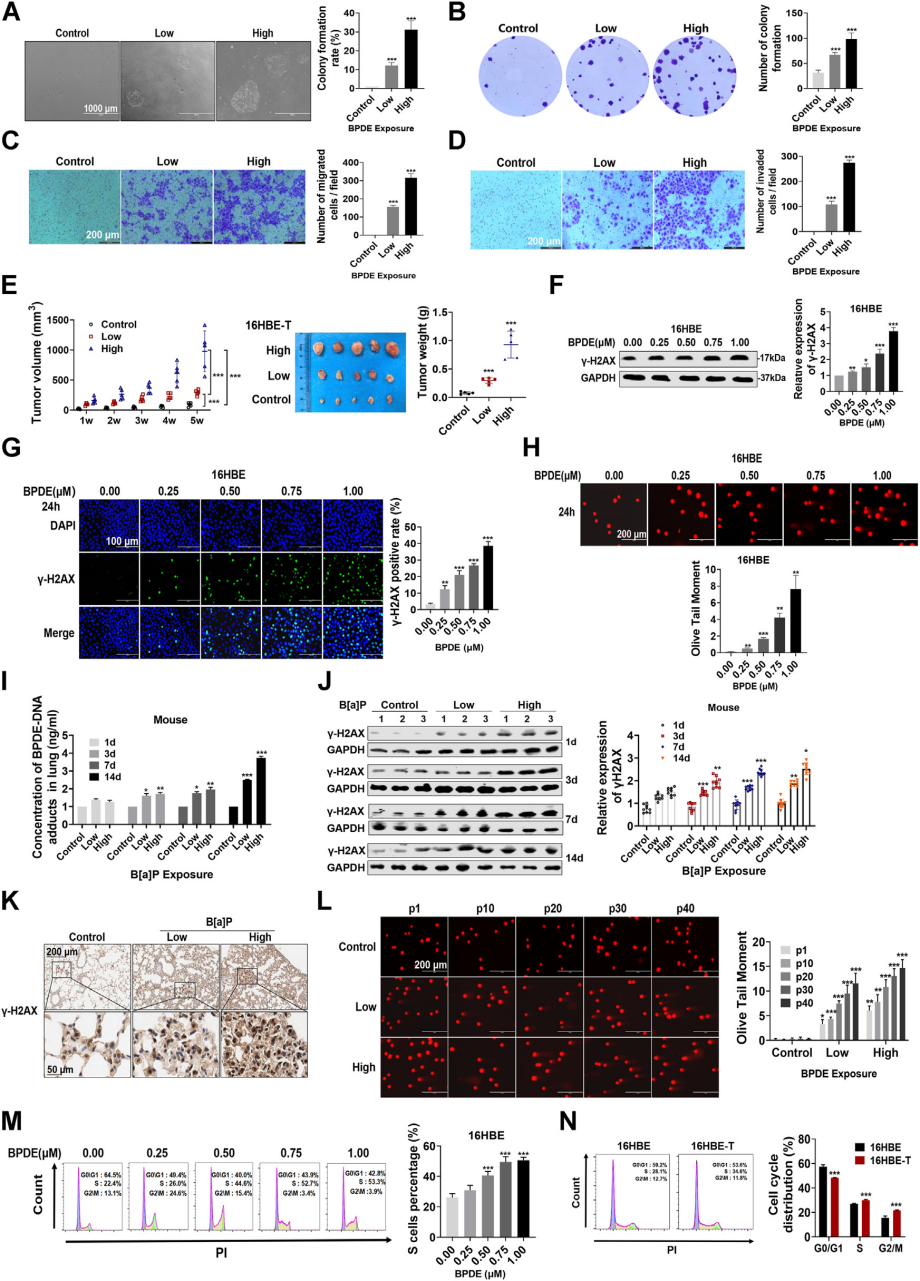
**DNA damage and cell cycle arrest persist during BPDE-induced lung carcinogenesis.***A* and *B*, soft-agar clone formation (*A*) and plate cloning (*B*) assays were used to determine the colony-forming ability of BPDE-transformed 16HBE cells (divided into control, low-, and high-dose groups). *C* and *D*, migratory (*C*) and invasive (*D*) capacities of BPDE-transformed cells. *E*, validation of BPDE-induced malignant transformation of 16HBE cells in a nude mouse xenograft model. *F*, protein expression of γ-H2AX in 16HBE cells after 24 h of exposure to different concentrations of BPDE. *G*, immunofluorescence detection of the expression of γ-H2AX in 16HBE cells after 24 h of exposure to different concentrations of BPDE. *H*, single cell gel electrophoresis assay for the detection of DNA damage in 16HBE cells treated with different concentrations of BPDE for 24 h. *I*, ELISA detection of BPDE-DNA adducts in the lung tissues of A/J mice exposed to B[a]P. *J* and *K*, Western blotting (*J*) and immunohistochemistry (*K*) were performed to detect γ-H2AX expression in the lung tissues of A/J mice exposed to B[a]P. *L*, quantification of DNA damage in different generations of 16HBE cells chronically exposed to BPDE using single-cell gel electrophoresis. *M*, cell cycle progression in 16HBE cells detected by flow cytometry after 24 h of exposure to different concentrations of BPDE. *N*, assessment of cell cycle progression in BPDE-transformed cells. Data represent mean ± SD from ≥3 biological replicates. Statistical significance was determined by Student's *t* test (two groups) or one-way ANOVA (≥3 groups): ∗*p* < 0.05, ∗∗*p* < 0.01, ∗∗∗*p* < 0.001.

A model of human bronchial epithelium malignancy was established through continuous exposing of 16HBE cells to BPDE, the most potent genotoxic and carcinogenic end-product of benzo(a)pyrene. The cytotoxicity of BPDE was determined using the cell counting kit-8 (CCK-8) and lactate dehydrogenase (LDH) assays before the exposure experiments. The results demonstrated that BPDE at concentrations close to 1 μM exhibited a significant cytotoxic effect on both the 16HBE and BEAS-2B cell lines (Fig. S1*A*). Therefore, we selected a maximum dose of 1 μM BPDE for subsequent experiments. The cellular model was established after 40 consecutive cycles of exposure and passage. The transformed cells showed abnormal morphology under the microscope (Fig. S1*B*). The results of the ethynyl-2′-deoxyuridine (EdU) assay showed that BPDE exposure increased bronchial epithelial cell proliferative activity in a time-dependent manner (Fig. S1*C*). Single-cell colony formation and nonanchored growth were analyzed in transformed cells using colony formation and soft agar cloning assays. Transformed cells in both high-dose (0.50 μM) and low-dose (0.25 μM) groups showed good single-cell colony forming and nonanchored growth abilities (Fig. 1, *A* and *B*). The migration and invasion of transformed cells were measured using Transwell assays. The results showed significant migration and invasion capacities in the two groups of transformed cells (Fig. 1, *C* and *D*). Similar results were obtained in the wound-healing assays (Fig. S1*D*). In a nude mouse xenograft model (Fig. S1*E*), subcutaneous tumor volume and weight were significantly higher in the high-dose or the low-dose BPDE-treated group than in the control group (Fig. 1*E*). These results suggest that long-term exposure to low doses of BPDE causes malignant transformation in 16HBE cells.

To investigate the early events in lung carcinogenesis, we first examined the expression of the γ-H2AX protein, which plays a role in DNA damage recognition (19). Western blot analysis showed that BPDE exposure upregulated γ-H2AX levels in a dose-dependent manner (Fig. 1*F*). Immunofluorescence assays confirmed these results, showing a significant increase in γ-H2AX positivity in response to BPDE treatment (Fig. 1*G*). Consistently, single-cell gel electrophoresis assays showed a trend towards DNA damage, with a significant increase in the olive tail moment (OTM) after BPDE exposure (Fig. 1*H*). Similar results showing BPDE-induced DNA damage were obtained in BEAS-2B cells (Fig. S1, *F*–*H*). In A/J mice with short-term B[a]P exposure (Fig. S1*I*), hematoxylin and eosin (HE) staining indicated worsening inflammatory cell infiltration and lung tissue edema with increasing exposure number and dose (Fig. S1*J*). ELISA analysis of mouse lung tissues showed a time- and dose-dependent increase in the levels of BPDE-DNA adducts (Fig. 1*I*), whereas immunoblotting and immunohistochemistry assays showed upregulation of γ-H2AX (Fig. 1, *J* and *K*). During the malignant transformation of 16HBE cells, BPDE-induced DNA damage persisted and worsened in correlation with increasing transformation stages (Fig. 1*L*). Cell cycle progression, the alteration of which is an indicator of DNA damage, was examined by flow cytometry, which showed a dose-dependent increase in the percentage of cells in S-phase in response to BPDE (Fig. 1*M*). These changes in cell cycle distribution were also observed in 16HBE-T cells undergoing malignant transformation (Fig. 1*N*).

These results suggest that continuous BPDE exposure led to the malignant transformation of bronchial epithelial cells. BPDE caused DNA damage and cell cycle arrest, and these changes persisted in cells that underwent malignant transformation.

### Lung cancer-associated circ_0004470 is upregulated in response to BPDE exposure and promotes BPDE-induced lung carcinogenesis

**Figure 2:**
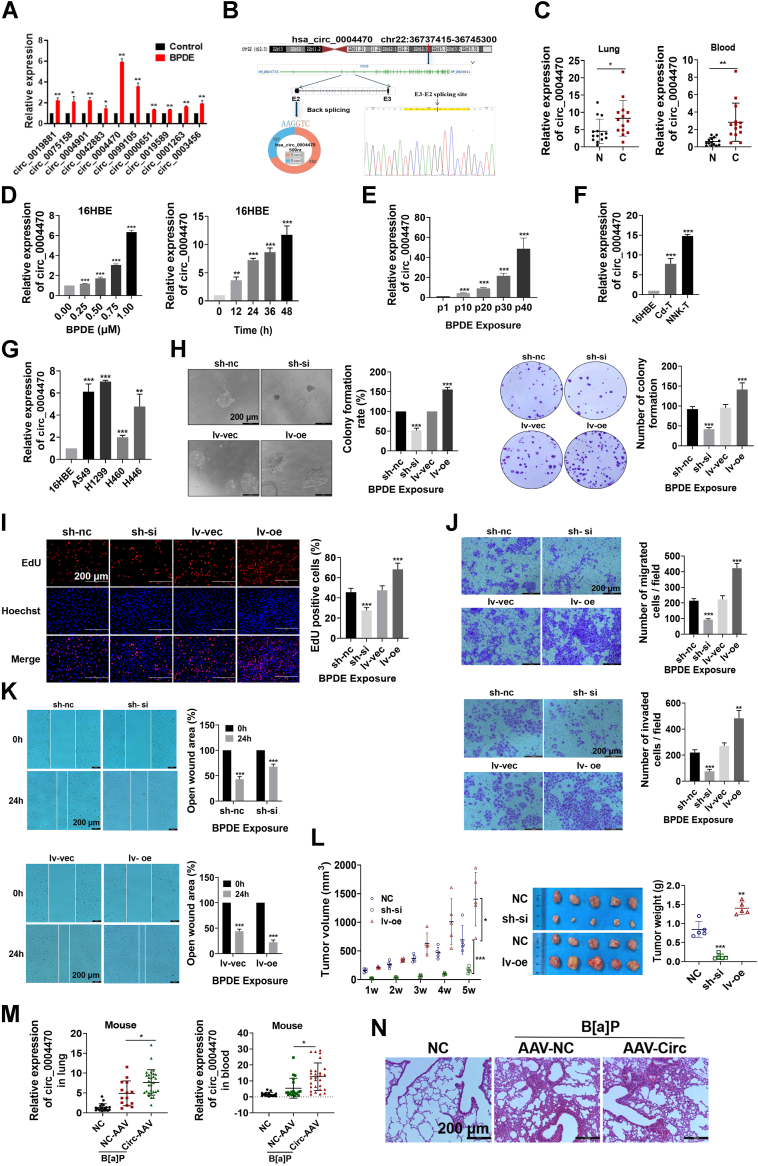
**Lung cancer–associated circ_0004470 is upregulated after BPDE exposure and promotes BPDE-induced lung carcinogenesis**. *A*, circular RNA differential expression profiles in a model of BPDE-induced DNA damage analyzed by qRT-PCR. *B*, schematic structure of circ_0004470 and Sanger sequencing results. *C*, expression of circ_0004470 in lung cancer tissues and blood samples from clinical patients. *D*, expression of circ_0004470 in 16HBE cells exposed to BPDE at different concentrations and times. *E*, qRT-PCR analysis of the expression of circ _ 0004470 in different generations of 16HBE cells chronically exposed to BPDE. *F* and *G*, expression of circ_0004470 in Cd- or NNK-transformed cells (*F*) and in the lung cancer A549, H299, H446, and H460 cell lines (*G*). *H* and *I*, effect of circ_0004470 on the malignant proliferative capacity of BPDE-transformed cells assessed using soft-agar clone formation and plate-cloning assays (*H*) and EdU assays (*I*). *J* and *K*, transwell (*J*) and wound-healing (*K*) assays of the effect of circ_0004470 on the migratory and invasive capacities of 16HBE cells transformed by BPDE. *L*, a nude mice xenograft model was used to evaluate the effect of circ_0004470 on the tumorigenic capacity of BPDE-transformed cells. *M*, circ_0004470 expression in lung tissue and blood of A/J mice chronically exposed to B[a]P. *N*, HE staining for the assessment of pathological changes in the lungs of A/J mice chronically exposed to B[a]P with high expression of circ_0004470. Data represent mean ± SD from ≥3 biological replicates. Statistical significance was determined by Student's *t* test (two groups) or one-way ANOVA (≥3 groups):∗*p* < 0.05, ∗∗*p* < 0.01, ∗∗∗*p* < 0.001.

To identify differentially expressed circRNAs associated with bronchial epithelial cell carcinogenesis, the circRNA expression profile was analyzed using whole transcriptome sequencing (Fig. S2, *A* and *B*). Quantitative real-time PCR (qRT-PCR) analysis confirmed that multiple circRNAs were upregulated in BPDE-exposed cells, of which circ_0004470 (circBase ID: hsa_circ_0004470) showed 5.94-fold upregulation (Fig. 2*A*). Circ_0004470 was prioritized for further study due to its highest upregulation magnitude, clinical relevance in lung cancer samples, and robust functional validation across carcinogen models. Circ_0004470 is formed by reverse splicing of the exon2 and exon3 transcripts of the parental gene MYH9, located at chr22:36737415-36745300, and Sanger sequencing confirmed the specific back-splicing sequence of circ_0004470 (Fig. 2*B*). The stable circular structure of circ_0004470 was confirmed by actinomycin D (AD) and ribonuclease R resistance assays (Fig. S2*C*). Fluorescence *in situ* hybridization (Fig. S2*D*) and qRT-PCR after nucleo-cytoplasmic separation (Fig. S2*E*) showed that circ_0004470 was mainly located in the cytoplasm. Further qRT-PCR analysis showed that circ_0004470 was upregulated in the tissues and blood of lung cancer patients, suggesting that circ_0004470 is a potential biomarker for lung cancer (Fig. 2*C*).

The potential role of circ_0004470 in lung carcinogenesis was investigated by analyzing the dose- and time-dependent relationship between BPDE exposure and circ_0004470. qRT-PCR showed that BPDE upregulated circ_0004470 in a dose- and time-dependent manner in 16HBE and BEAS-2B cells (Figs. 2*D* and S2*F*). In BPDE-induced carcinogenesis, circ_0004470 expression increased with increasing passages (Fig. 2*E*). Circ_0004470 upregulation was not only observed in relation to BPDE-induced carcinogenesis but also in cells undergoing malignant transformation in response to the carcinogen NNK and the heavy metal cadmium (Fig. 2*F*), and in the canonical lung cancer cell lines A549, H1299, H460, and H446 (Fig. 2*G*). Taken together, these results suggest that circ_0004470 plays an important role in the initiation of lung cancer.

To investigate the role of circ_0004470 in lung cancer initiation, we constructed circ_0004470 stable overexpression and knockdown 16HBE cells (Fig. S2*G*). After 40 passages with continuous BPDE treatment, clonogenic and EdU assays indicated that all cells transformed by BPDE exposure acquired malignant proliferation ability (Fig. 2, *H* and *I*). The malignant proliferative capacity of the circ_0004470 overexpression group was significantly higher than that of the respective control group, whereas that of the knockdown group was significantly inhibited. The results of Transwell assays indicated that circ_0004470 overexpression significantly promoted, whereas circ_0004470 knockdown significantly inhibited the migration and invasion of BPDE-transformed cells (Fig. 2*J*). Similar results were obtained in wound-healing assays (Fig. 2*K*). *In vivo* xenograft experiments, subcutaneous tumors formed by circ_0004470-overexpressing transformed cells were larger, whereas those in the low-expression group were smaller than those of the respective control groups (Fig. 2*L*).

In the B[a]P-induced lung cancer model in A/J mice, circ_0004470 expression was increased in lung tissue and blood in the exposed group. Pre-injection of circ_0004470-overexpressing adenovirus resulted in BPDE-induced circ_0004470 overexpression in the lungs and blood of A/J mice (Fig. 2*M*). HE staining showed that lung tissue lesions and focal alveolar epithelial hyperplasia were more severe in the lungs of mice overexpressing circ_0004470 than in the controls (Fig. 2*N*). Taken together, these results indicate that circ_0004470 may promote lung carcinogenesis both *in vitro* and *in vivo*.

### Circ_0004470 promotes DNA damage and suppresses cell cycle progression in BPDE-treated cells

**Figure 3:**
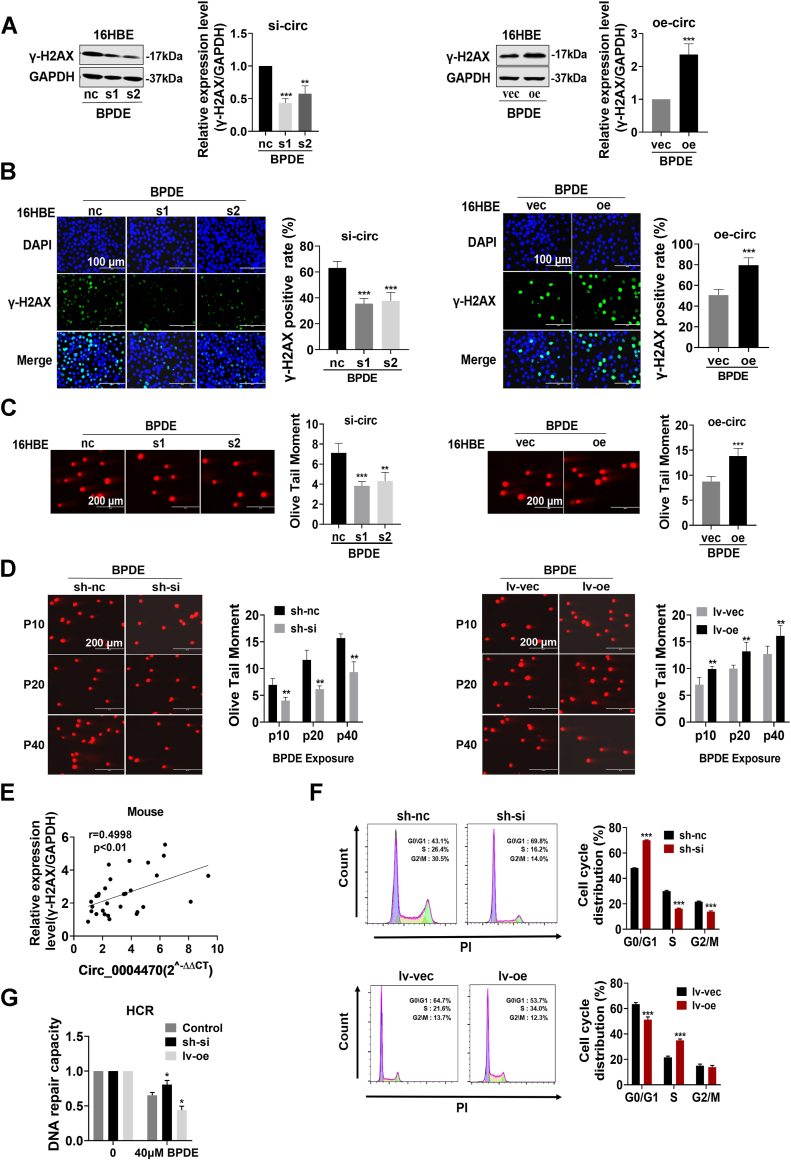
**Circ_0004470 promotes DNA damage and suppresses cell cycle progression in BPDE-treated cells.***A*, Western blot detection of the effect of circ_0004470 on γ-H2AX expression in BPDE-treated 16HBE cells. *B*, immunofluorescence analysis of the effect of circ_0004470 on γ-H2AX expression in BPDE-treated 16HBE cells. *C*, effect of circ_0004470 on DNA damage in 16HBE cells upon acute exposure to BPDE analyzed by single cell gel electrophoresis. *D*, SCGE experiments to monitor the effect of circ_0004470 on DNA damage in 16HBE cells transformed by long-term exposure to BPDE. *E*, correlation between circ_0004470 and γ-H2AX in the lung tissues of A/J mice after short-term repeated exposure to B[a]P. *F*, effects of circ_000447 on cell cycle progression in BPDE-transformed cells determined by flow cytometry. *G*, the host cell reactivation assay was performed to evaluate the impact of circ_000447 on the DNA repair capacity of cells that underwent BPDE-mediated malignant transformation cells. Data represent mean ± SD from ≥3 biological replicates. Statistical significance was determined by Student's *t* test (two groups) or one-way ANOVA (≥3 groups): ∗*p* < 0.05, ∗∗*p* < 0.01, ∗∗∗*p* < 0.001.

Because DNA damage is a crucial step in carcinogenesis (20), we designed a system for functional analysis by modulating the expression of circ_0004470 using siRNAs and overexpression plasmids. This system can significantly alter circ_0004470 expression without affecting the parental gene MYH9 (Fig. S3*A*). In addition, BPDE-exposed cells showed normal function of the circ_0004470 system (Fig. S3*B*). Western blot analysis showed that knockdown of circ_0004470 significantly decreased the expression of γ-H2AX induced by BPDE, whereas overexpression of circ_0004470 promoted the expression of γ-H2AX (Fig. 3*A*). Immunofluorescence analysis showed similar results, with a significant decrease in γ-H2AX–positive cells in the circ_0004470 knockdown group and an increase in BPDE-induced γ-H2AX–positive cells in the overexpression group (Fig. 3*B*). Single-cell gel electrophoresis assays showed that silencing circ_0004470 decreased the OTM value, whereas early overexpression of circ_0004470 significantly increased BPDE-induced OTM (Fig. 3*C*). The results observed in BPDE-exposed BEAS-2B cells were consistent with those in 16HBE cells (Fig. S3, *C* and *D*). Long-term exposure to BPDE had different effects on DNA damage in 16HBE cells with stable knockdown or overexpression of circ_0004470 (Figs. 3*D* and S3*E*). *In vivo*, a positive correlation between circ_0004470 and γ-H2AX was observed in mice subjected to short-term repetitive B[a]P exposure (Fig. 3*E*). These data indicate that circ_0004470 may contribute to BPDE-induced DNA damage.

Next, we investigated the potential regulatory role of circ_0004470 in the cellular DDR. BPDE-induced S phase arrest was attenuated in malignant cells with circ_0004470 knockdown and increased in those with circ_0004470 overexpression compared with the control groups (Fig. 3*F*). Host cell reactivation assays showed that persistent BPDE exposure impaired the DNA repair capacity of normal 16HBE cells. Overexpression of circ_0004470 significantly aggravated the impairment of DNA repair in BPDE-transformed cells, whereas circ_0004470 knockdown partially restored this ability (Fig. 3*G*).

Taken together, these results suggest that circ_0004470 plays a role in BPDE-induced DNA damage. Circ_0004470 may promote the accumulation of unrepaired damaged DNA by regulating cell cycle distribution and by inhibiting DNA repair in the process of BPDE-induced lung carcinogenesis.

### Circ_0004470 targets XPC and disrupts the XPC–hHR23B repair complex

**Figure 4:**
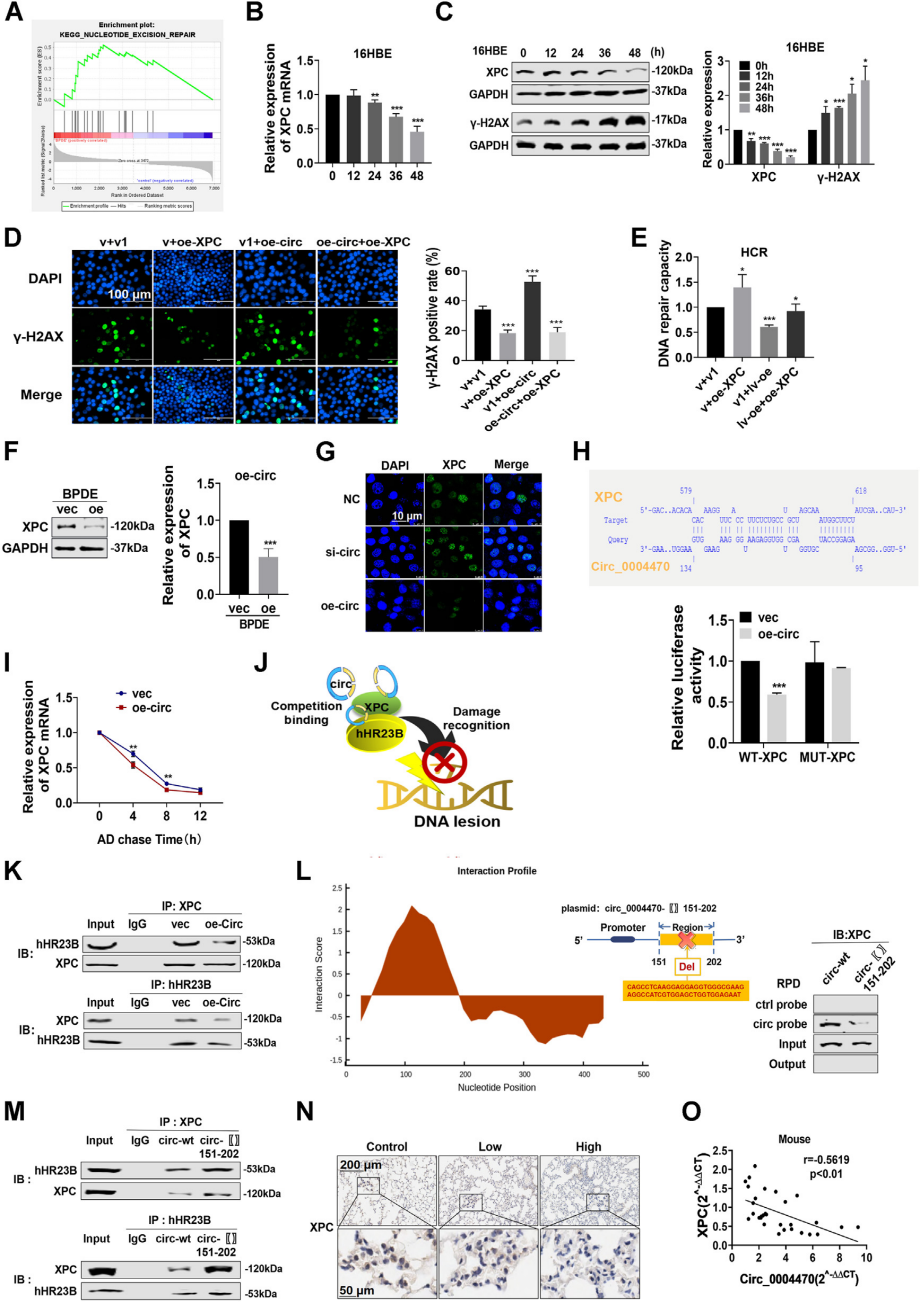
**Circ_0004470 targets XPC and disrupts the XPC–hHR23B repair complex.***A*, gene set enrichment analysis revealed aberrations in the nucleotide excision repair pathway. *B*, XPC mRNA expression in 16HBE cells exposed to 1 μM BPDE for different periods. *C*, protein expression of XPC and γ-H2AX in 16HBE cells exposed to 1 μM BPDE at different times. *D* and *E*, immunofluorescence (*D*) and host cell reactivation assays (*E*) were used to evaluate the effect of circ_0004470 and XPC cotransformation on DNA damage and repair in 16HBE cells. *F*, effect of circ_0004470 overexpression combined with BPDE exposure on XPC protein expression. *G*, effect of circ_0004470 knockdown or overexpression combined with BPDE exposure on the nucleoplasmic distribution of XPC. *H*, dual luciferase assays were used to validate the potential binding regions of circ_0004470 and XPC mRNA as predicted by IntaRNA. *I*, effect of circ_0004470 overexpression on the stability of XPC mRNA in actinomycin D–treated 16HBE cells. *J*, hypothetical plot of the effect of circ_0004470 on XPC and hHR23B dimer formation under BPDE exposure. *K*, co-immunoprecipitation experiments confirmed the competitive binding of overexpressed circ_0004470 to XPC-hHR23B. *L*, RNA pull-down was used to validate the potential binding region of circ_0004470 to the XPC protein as predicted by the CatRAPID database. *M*, co-immunoprecipitation experiments were performed to compare the effect of transfecting WT or mutant plasmids containing circ_0004470 and the XPC protein interaction regions on XPC-hHR23B binding. *N*, XPC protein expression in the lung tissues of A/J mice after short-term repeated exposure to B[a]P. *O*, correlation of XPC mRNA with circ_0004470 in the lung tissues of A/J mice after short-term repeated exposure to B[a]P. Data represent mean ± SD from ≥3 biological replicates. Statistical significance was determined by Student's *t* test (two groups) or one-way ANOVA (≥3 groups): ∗*p* < 0.05, ∗∗*p* < 0.01, ∗∗∗*p* < 0.001.

To elucidate the mechanism underlying the role of circ_0004470 in the DDR during BPDE-induced lung carcinogenesis, we performed pathway enrichment analysis to identify altered pathways in BPDE-exposed cells. Gene set enrichment analysis demonstrated that the NER pathway was significantly enriched and correlated with BPDE exposure (Fig. 4*A*). Additionally, gene set enrichment analysis identified XPC as a core gene of the NER pathway. XPC is a vital component of the NER pathway that recognizes and binds to DNA damage sites through the formation of XPC protein complexes (21). The results of qRT-PCR showed that BPDE exposure downregulated XPC in a time-dependent manner (Fig. 4*B*). Western blot analysis showed a gradual decline in XPC protein levels in correlation with increasing BPDE-induced DNA damage (Fig. 4*C*). In BPDE-exposed cells, XPC was progressively downregulated with increasing transformation passages both at the transcriptional and translational levels (Fig. S4, *A* and *B*). We therefore hypothesized that XPC is important for BPDE-induced carcinogenesis.

To prove this hypothesis, we constructed XPC overexpression plasmids to determine whether XPC could rescue BPDE-induced DNA damage (Fig. S4*C*). Overexpression of XPC significantly decreased the number of BPDE-damaged cells (Fig. S4*D*) and increased the DNA repair capacity (Fig. S4*E*). Cotransfection experiments (Fig. 4, *D* and *E*) showed that XPC overexpression significantly impaired the effect of circ_0004470 on inducing DNA damage and inhibiting DNA repair in BPDE-exposed cells. Western blot analysis showed that circ_0004470 overexpression markedly downregulated XPC expression (Fig. 4*F*), whereas circ_0004470 knockdown had the opposite effect (Fig. S4*F*). Immunofluorescence staining revealed a significant decrease in intranuclear XPC protein expression in cells with circ_0004470 overexpression combined with BPDE exposure, whereas the knockdown group showed the opposite pattern (Fig. 4*G*). To examine the underlying mechanism, the potential binding site between circ_0004470 and XPC mRNA was predicted using IntaRNA webtools (rna.informatik.uni-freiburg.de/IntaRNA) and verified by luciferase reporter gene assays (Fig. 4*H*). Upon AD treatment, XPC mRNA degradation was accelerated by circ_0004470 overexpression (Fig. 4*I*), indicating that circ_0004470 can directly bind to XPC mRNA and affect its stability. In the context of DNA damage, XPC forms a dimer with hHR23B to identify the DNA damage site and initiate the NER process (21). We therefore hypothesized that circ_0004470 might interfere with XPC and hHR23B dimer formation upon DNA damage (Fig. 4*J*). This hypothesis was confirmed by co-IP assays: circ_0004470 overexpression inhibited XPC-precipitated hHR23B, as supported by reverse precipitation using an hHR23B antibody (Fig. 4*K*). We used the CatRAPID database (http://service.tartaglialab.com/) to predict potential XPC-binding regions in circ_0004470, and the 151 to 202 bp region showed the highest score (Fig. 4*L*, left). RNA pulldown using a biotin-labeled circ_0004470 probe with or without the predicted binding region confirmed that this region was the XPC-binding site (Fig. 4*L*, right). Deletion of the binding fragment abolished the inhibitory effect of circ_0004470 on the XPC–hHR23B interaction, as shown by co-IP assay (Fig. 4*M*). *In vivo*, immunohistochemistry demonstrated a significant reduction in XPC expression in the lungs of B[a]P-exposed mice (Fig. 4*N*), and qRT-PCR of mouse lung tissues indicated a negative correlation between circ_0004470 and XPC expression (Fig. 4*O*).

Taken together, these data suggest that circ_0004470 regulates BPDE-induced DNA damage and NER suppression by controlling XPC degradation and the XPC–hHR23B protein interaction.

### Circ _ 0004470 promotes lung progression by inhibiting XPC

**Figure 5:**
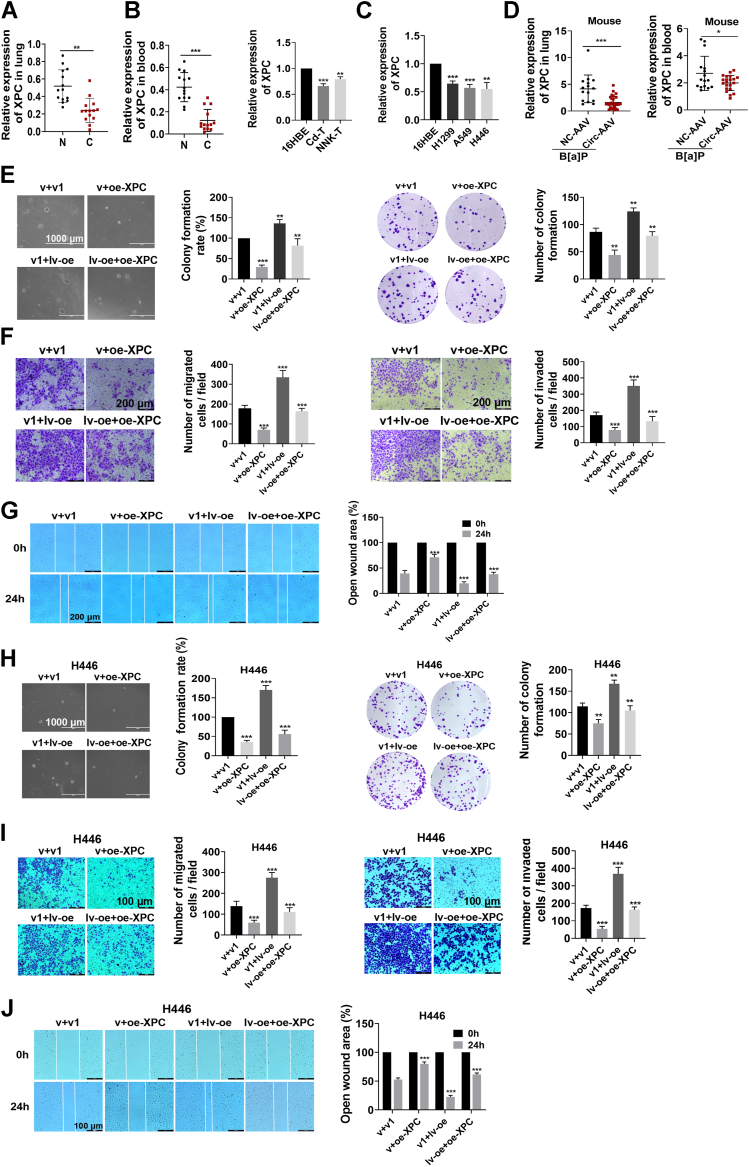
**Circ _ 0004470 promotes lung progression by inhibiting XPC.***A*, expression of XPC mRNA in lung tissues and blood samples from clinical cases. *B*, XPC mRNA expression in cells transformed by Cd or NNK. *C*, Expression of XPC mRNA in H1299, A549, and H446 cells. *D*, XPC mRNA expression in the lungs and blood of A/J mice subjected to chronic B[a]P exposure. *E*, soft agar clone formation and plate cloning assays were used to evaluate the effect of cotransfection with XPC and circ_0004470 on the malignant proliferative capacity of BPDE-transformed cells. *F* and *G*, transwell (*F*) and wound-healing (*G*) assays were performed to evaluate the effect of cotransfection of XPC with circ_0004470 on the migratory and invasive abilities of BPDE-transformed cells. *H*, soft agar clone formation and plate cloning assays were used to evaluate the effect of cotransfection with circ_0004470 and XPC on the proliferation of H446 cells. *I* and *J*, transwell (*I*) and wound-healing (*J*) assays were used to assess the effect of cotransfection with circ_0004470 and XPC on the metastatic ability of H446 cells. Data represent mean ± SD from ≥3 biological replicates. Statistical significance was determined by Student's *t* test (two groups) or one-way ANOVA (≥3 groups): ∗*p* < 0.05, ∗∗*p* < 0.01, ∗∗∗*p* < 0.001.

The XPC protein exerts anticancer effects in lung tumors by inhibiting cell proliferation and metastasis (22). Low XPC expression is associated with decreased overall survival in lung cancer patients, according to a public database Kaplan–Meier analysis (Fig. S5*A*). We found that XPC expression was significantly lower in the lungs and blood of lung cancer patients than in those of the controls (Fig. 5*A*). XPC mRNA expression was also downregulated in NNK- and Cd-induced malignant cells (Fig. 5*B*) and lung cancer cell lines (A549, H1299, and H446) (Fig. 5*C*) and even lower in the lungs and blood of mice with high circ_0004470 expression (Fig. 5*D*).

A previous study confirmed the role of circ_0004470 in lung carcinogenesis. To determine whether circ_0004470 regulates the function of lung cancer cells through XPC, we performed cotransfection experiments. In BPDE-transformed cells, XPC overexpression significantly inhibited cell proliferation and restored the pro-proliferative effect of circ_0004470, as demonstrated by colony formation and EdU assays (Figs. 5*E* and S5*B*). Transwell and wound-healing assays demonstrated that XPC overexpression markedly inhibited migration and invasion and impaired the ability of circ_0004470 to promote malignant metastasis of transformed cells (Fig. 5, *F* and *G*). In cancer cells, XPC overexpression suppressed the proliferative and metastatic capacities of H446 lung cancer cells and abrogated the effects of circ_0004470 on promoting proliferation and metastasis (Fig. 5, *H*–*J*). Similar results were obtained in A549 and H1299 cells (Fig. S5, *C*–*G*). The results indicate that XPC plays a crucial role in the effect of circ_0004470 on promoting lung carcinogenesis.

### Circ_0004470 interferes with cell cycle regulation *via* DDB1

**Figure 6:**
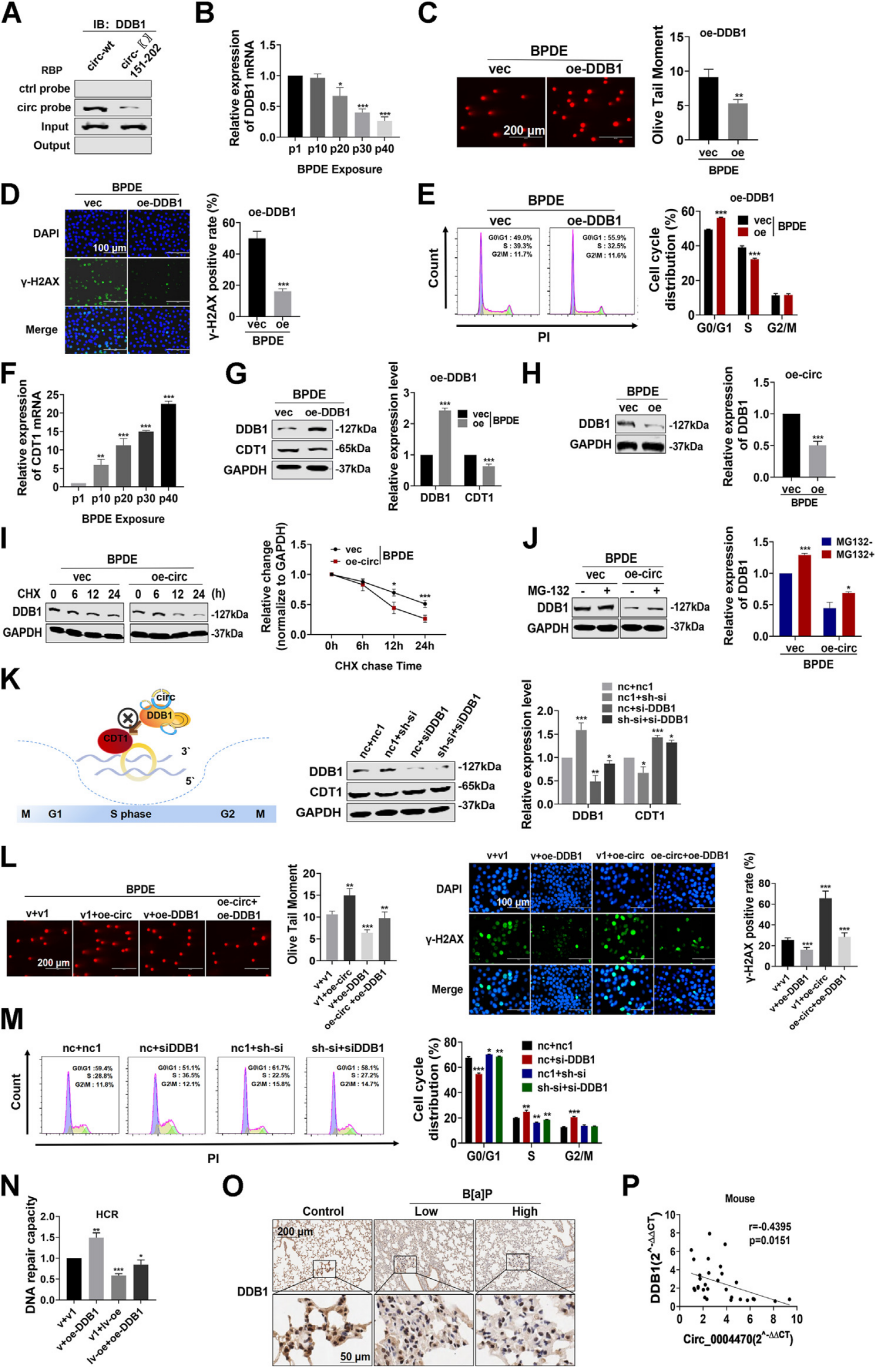
**Circ_0004470 interferes with cell cycle regulation *via* DDB1.***A*, RNA pull-down assays were used to validate the ability of circ_0004470 to enrich for the DDB1 protein. *B*, expression of DDB1 mRNA in the different generations of 16HBE cells chronically exposed to BPDE. *C* and *D*, single-cell gel electrophoresis (*C*) and immunofluorescence (*D*) assays were used to assess the effect of DDB1 overexpression on BPDE-induced DNA damage in 16HBE cells. *E*, effect of DDB1 overexpression on the cell cycle of 16HBE cells exposed to BPDE. *F*, expression of CDT1 mRNA in the different generations of 16HBE cells chronically exposed to BPDE. *G*, effect of DDB1 overexpression on CDT1 protein expression in BPDE-exposed 16HBE cells. *H*, effect of circ_0004470 overexpression combined with BPDE exposure on DDB1 protein expression. *I*, effect of circ_0004470 on the half-life of the DDB1 protein after actinomycin treatment combined with BPDE exposure. *J*, effect of circ_0004470 overexpression combined with BPDE exposure on the stability of the DDB1 protein in the presence or absence of the proteasome inhibitor MG132. *K*, hypothetical diagram of the effect of circ_0004470 on disrupting cell cycle progression by interfering with CDT1 regulation by DDB1 upon DNA damage (*left*) and the effect of circ_0004470 cotransfection with DDB1 on CDT1 protein expression (*right*). *L*, single-cell gel electrophoresis and immunofluorescence assays were used to evaluate the effect of cotransfection with circ_0004470 and DDB1 on BPDE-induced DNA damage in 16HBE cells. *M*, effect of circ_0004470 cotransfection with DDB1 on the cell cycle of BPDE-transformed 16HBE cells. *N*, host cell reactivation assays were used to evaluate the impact of circ_0004470 cotransfection with DDB1 on the DNA repair capacity of BPDE-transformed cells. *O*, immunohistochemical analysis of DDB1 expression in lung tissues of A/J mice after short-term repeated exposure to B[a]P. *P*, correlation of DDB1 mRNA with circ_0004470 in the lung tissues of A/J mice subjected to short-term repeated exposure to B[a]P. Data represent mean ± SD from ≥3 biological replicates. Statistical significance was determined by Student's *t* test (two groups) or one-way ANOVA (≥3 groups): ∗*p* < 0.05, ∗∗*p* < 0.01, ∗∗∗*p* < 0.001.

RNA pulldown using a circ_0004470-specific probe identified DDB1 as an interacting RNA-binding protein (Fig. S6*A*). DDB1 plays a crucial role in NER by regulating cell cycle progression (14). Further analysis identified the 151 to 202 bp region as the potential site for the circ_0004470–DDB1 interaction (Fig. 6*A*). The interaction between DDB1 and circ_0004470 was validated by RNA immunoprecipitation assays using a DDB1-specific antibody (Fig. S6*B*). During BPDE-induced cell transformation, DDB1 was downregulated at the transcriptional and translational levels in correlation with an increasing number of passages (Figs. 6*B* and S6*C*). Because this suggested that DDB1, an interacting RBP of circ_0004470, was involved in BPDE-induced carcinogenesis, we established DDB1 knockdown and overexpression systems to perform functional analyses (Fig. S6*D*). The results of single-cell gel electrophoresis and immunofluorescence staining assays demonstrated that DDB1 overexpression inhibited BPDE-induced DNA damage (Fig. 6, *C* and *D*), whereas DDB1 knockdown had the opposite effect (Fig. S6*E*). Flow cytometry showed that overexpression of DDB1 significantly inhibited BPDE-induced S-phase arrest (Fig. 6*E*) whereas DDB1 knockdown aggravated S-phase arrest (Fig. S6*F*). These results indicate that DDB1 is involved in the regulation of BPDE-induced DNA damage and cell cycle S phase arrest.

Chromatin licensing and DNA replication factor 1 (CDT1), a cell cycle–regulated partner of DDB1, is upregulated in several early precancerous lesions in lung cancer (23). Kaplan–Meier analysis from a public database indicated that high CDT1 expression is associated with lower overall survival in lung cancer patients (Fig. S6*G*). Long-term exposure of 16HBE cells to BPDE resulted in CDT1 upregulation in successive generations (Fig. 6*F*), and overexpression of DDB1 downregulated CDT1 (Fig. 6*G*). Conversely, knockdown of DDB1 upregulated CDT1 (Fig. S6*H*). Taken together with the aforementioned cell cycle regulatory role of DDB1, these results suggest that the effect of DDB1 on BPDE-induced cell cycle S phase arrest is mediated by CDT1.

After clarifying the cell cycle regulatory effect of DDB1, the interacting protein of circ_0004470, we sought to understand how DDB1 is involved in the regulatory function of circ_0004470. Circ_0004470 overexpression caused a significant downregulation of DDB1 protein expression (Fig. 6*H*), whereas circ_0004470 knockdown upregulated DDB1 expression (Fig. S6*I*). Treatment with the protein synthesis inhibitor cycloheximide showed that circ_0004470 decreases the half-life of the DDB1 protein (Fig. 6*I*). By contrast, treatment with the proteasome inhibitor MG-132 reversed the effect of circ_0004470 on promoting DDB1 degradation (Fig. 6*J*). This led us to hypothesize that circ_0004470 interacts with DDB1 to regulate CDT1 expression in response to BPDE-induced DNA damage and cell cycle progression. To verify this hypothesis, we performed cotransfection experiments, which demonstrated that CDT1 protein levels are coregulated by both circ_0004470 and DDB1 (Fig. 6*K*). Subsequent functional studies in cells cotransfected with circ_0004470 and DDB1 showed that DDB1 overexpression significantly impaired the effect of circ_0004470 on inducing DNA damage (Fig. 6*L*), promoting the S phase of cell division (Fig. 6*M*), and inhibiting DNA repair in BPDE-exposed cells (Fig. 6*N*). *In vivo*, immunohistochemistry demonstrated a significant reduction in DDB1 expression in the lungs of B[a]P-exposed mice (Fig. 6*O*). qRT-PCR analysis of the lung tissues of mice indicated a negative correlation between circ_0004470 and DDB1 expression (Fig. 6*P*).

The collective data indicate that circ_0004470 regulates BPDE-induced DNA damage and cell cycle arrest at S phase by interacting with DDB1 to regulate the expression of CDT1, a vital cell cycle regulator involved in NER.

### Circ_0004470 promotes lung cancer progression by inhibiting DDB1

**Figure 7:**
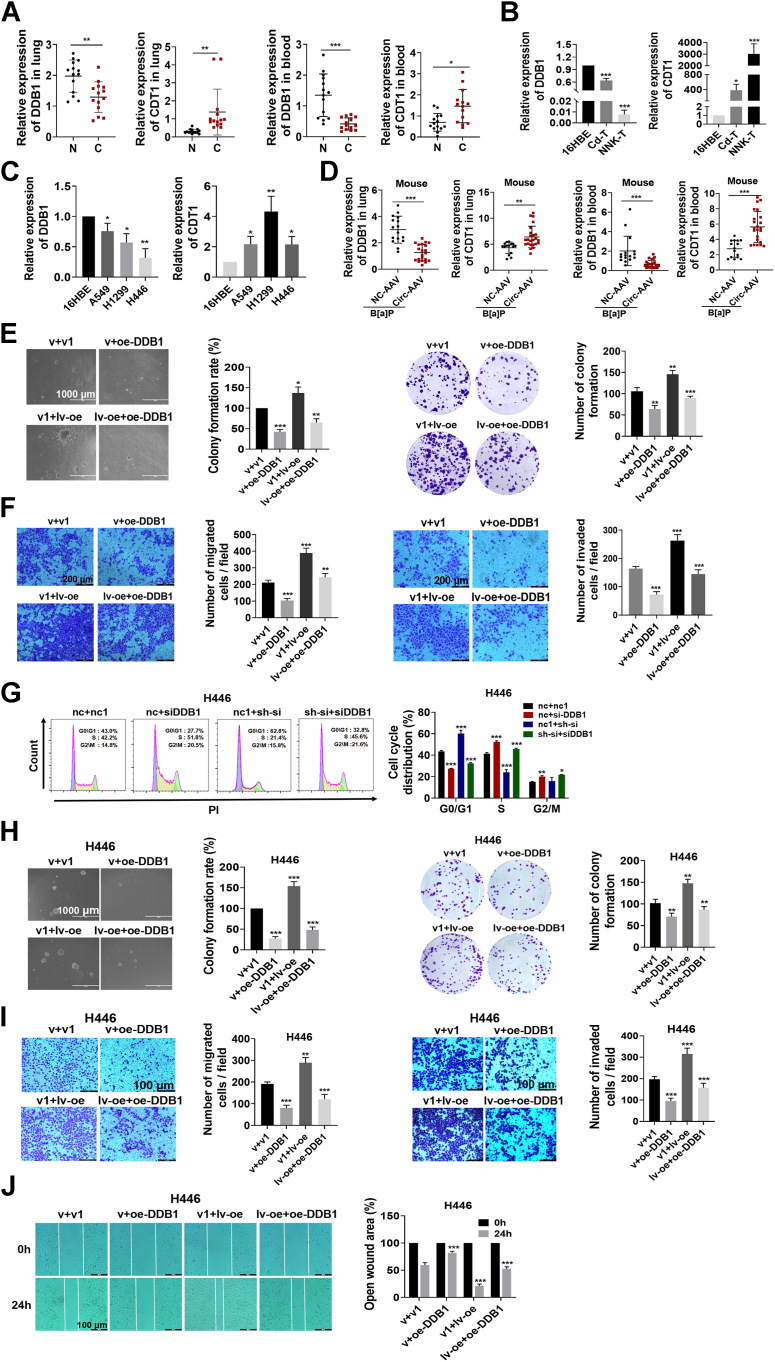
**Circ_0004470 promotes lung cancer progression by inhibiting DDB1**. *A*, mRNA expression of DDB1 and CDT1 in lung tissues and blood samples from clinical cases. *B*, mRNA expression of DDB1 and CDT1 in Cd- or NNK-transformed cells. *C*, mRNA expression of DDB1 and CDT1 in H1299, A549, and H446 cells. *D*, mRNA expression of DDB1 and CDT1 in the lung tissues and blood of chronically B[a]P-exposed A/J mice. *E*, soft agar clone formation and plate-cloning assays were used to evaluate the effect of cotransfection of DDB1 with circ_0004470 on the malignant proliferative capacity of BPDE-transformed cells. *F*, transwell assays were performed to evaluate the effect of cotransfection of DDB1 with circ_0004470 on the migratory and invasive abilities of BPDE-transformed cells. *G*, flow cytometry was used to determine the effect of cotransfection of circ_0004470 with DDB1 on the cell cycle of H446 cells. *H*, soft agar clone formation and plate-cloning assays were used to evaluate the effect of cotransfection of circ_0004470 with DDB1 on the proliferation of H446 cells. *I* and *J*, transwell (*I*) and wound-healing (*J*) assays were used to assess the effect of cotransfection of circ_0004470 with DDB1 on the metastatic ability of H446 cells. Data represent mean ± SD from ≥3 biological replicates. Statistical significance was determined by Student's *t* test (two groups) or one-way ANOVA (≥3 groups): ∗*p* < 0.05, ∗∗*p* < 0.01, ∗∗∗*p* < 0.001.

We demonstrated the significance of DDB1 and CDT1 in BPDE-induced DNA damage and cell cycle arrest, as well as their interaction with circ_0004470. Further studies are necessary to clarify the involvement of DDB1 and CDT1 in lung cancer progression, as well as their role in the regulation of circ_0004470 associated with lung cancer progression. Analysis of lung cancer clinical samples showed that the expression of DDB1 was significantly lower in lung cancer tissues than in normal tissues, whereas CDT1 was expressed at high levels in lung cancer tissues (Fig. 7*A*, left). Similar results were observed in the blood of lung cancer patients (Fig. 7*A*, right). In NNK- and Cd-transformed cells and lung cancer cell lines (A549, H1299, and H446), DDB1 was downregulated and CDT1 was upregulated (Fig. 7, *B* and *C*), consistent with the results in clinical samples. AAV-mediated circ_0004470 overexpression in mice downregulated DDB1 and upregulated CDT1 both in the lung and blood (Fig. 7*D*).

To elucidate the mechanism by which circ_0004470 regulates DDB1 during lung cancer progression, we performed cotransfection experiments. In BPDE-transformed cells, DDB1 overexpression significantly inhibited cell proliferation and reversed the proproliferative effect of circ_0004470, as determined by colony formation and EdU assays (Figs. 7*E* and S7*A*). Transwell and wound-healing assays demonstrated that DDB1 overexpression strongly inhibited migration and invasion and impaired the ability of circ_0004470 to promote the malignant metastasis of transformed cells (Figs. 7*F* and S7*B*). In Figure 6*M*, we demonstrated that DDB1 is a downstream target for the circ_0004470-mediated regulation of cell cycle arrest, and similar results were obtained in lung cancer cell lines. Flow cytometry analysis of cell cycle distribution in cotransfected cancer cells revealed that DDB1 inhibited the pro-S-phase blocking effects of circ_0004470 (Figs. 7*G* and S7*C*). DDB1 overexpression suppressed the proliferative and metastatic capacities of H446 cells and abrogated the effects of circ_0004470 on promoting proliferation and metastasis (Fig. 7, *H*–*J*). Similar results were obtained in A549 and H1299 cells (Fig. S7, *D*–*H*). In conclusion, we demonstrated the involvement of DDB1 in mediating the effect of circ_0004470 on lung cancer progression using multiple experimental models, including malignant transformation of cells, animal models, and clinical samples.

## Discussion

Genetic damage is a topic of great interest in cancer research, both in terms of the initiation of cancer and the development of effective cancer treatments (24). DNA is highly susceptible to damage caused by both internal and external factors. Among the factors leading to DNA damage, carcinogen-induced DNA damage is an important factor that requires comprehensive investigation (25). The accumulation of DNA damage is considered to be an important event in the process of carcinogenesis. For instance, lung carcinogenesis associated with PM10 exposure causes the accumulation of DNA damage due to inhibition of the NER pathway (26). A/C allele polymorphism in XPC is associated with smoking and may increase the risk of smoking-related oral cancer (27). PAHs are genotoxic chemicals that are commonly found in cigarette smoke and air pollutants. Several PAHs have been identified as risk factors for respiratory tumorigenesis, with B[a]P serving as the most representative carcinogen. *In vivo*, B[a]P is metabolically activated and exerts genotoxic effects on bronchial epithelial cells, resulting in the dysregulation of cellular function. Among the B[a]P activation products, BPDE is a potent genotoxic and carcinogenic factor that covalently binds to DNA to form stable adducts, which can lead to genetic mutations and ultimately result in the malignant transformation of normal cells and the development of cancer (28). In previous work from our group, we established a cellular carcinogenesis model by exposing bronchial epithelial cells to high doses of BPDE and observed that several oncogenes were upregulated in cells that underwent malignant transformation. We found that oncogenes such as c-myc and c-fos were highly expressed, whereas DNA repair genes such as XPC and RAD50 were downregulated (29). In this study, we used low doses of BPDE to induce DNA damage without causing lethal effects. Prolonged exposure to low-dose BPDE caused the malignant transformation of bronchial epithelial 16HBE cells concomitant with a sustained increase in DNA damage, a progressive decrease in DNA repair capacity, and cell cycle arrest at the S phase.

Normal cells are well equipped to handle DNA damage because they possess a sophisticated DDR system. The DDR consists of a series of coordinated cellular responses initiated by the cell in response to DNA damage. The DDR encompasses a spectrum of processes, including damage recognition, DNA repair, cell cycle arrest, and the regulation of different cellular outcomes. In the majority of cases, cells complete the repair process and resume normal cellular function. Alternatively, they may activate the programmed cell death system, which enables the timely clearance of cells with severe DNA damage. In rare cases, under the influence of multiple factors such as abnormal DNA repair systems and failure of the programmed cell death system, cells with damaged genomes survive and develop tumor characteristics such as abnormal proliferation and invasion ability, that is, carcinogenesis (30). NER, which is one of the most fundamental pathways of DNA repair, is divided into transcription-coupled repair and global genomic repair (31). XPC plays a role in the damage recognition process of global genomic repair, including the recognition of PAH-induced DNA adducts, UV-induced cyclobutene-pyrimidine dimers, and (6–4) pyrimidine-pyrimidinone dimers. This recognition occurs through the formation of dimers that interact with the hHR23B protein. XPC is downregulated in lung adenocarcinoma patients, and downregulation of XPC in lung cancer cells is associated with increased expression of cancer stem cell biomarkers and cell invasive capacity (32). Arrest of the cell cycle in response to DNA damage allows sufficient time for DNA repair. Hydrolysis of the permissive factor CDT1 following DNA damage is regarded as a checkpoint that ensures that there is sufficient time for repair prior to DNA replication. DDB1 functions as a junction protein for the E3 ubiquitin ligase Cullin4 complex. This complex promotes NER, thereby regulating DNA replication and mediating the hydrolysis of CDT1 proteins during the S phase or after DNA damage (13, 33). Cells depleted of DDB1 exhibit elevated levels of CDT1 protein and increased replication, resulting in the accumulation of DNA damage (33). Decreased expression of DDB1 is a risk factor for head and neck squamous cell carcinoma (34). In a mouse model, treatment with cigarette smoke extracts led to the degradation of proteins in lung epithelial cells, which was mediated by the DDB1 E3 ligase complex (35). In this study, we found that in 16HBE cells, both short- and long-term BPDE exposure downregulated XPC and DDB1 and upregulated CDT1. This resulted in the inhibition of the nucleotide repair pathway, a gradual accumulation and aggravation of BPDE-induced DNA damage, and a progressive arrest of the cell cycle in the S-phase. Ultimately, these changes led to the malignant transformation of normal bronchial epithelial cells.

Advances in biological research have highlighted the importance of epigenetic changes in carcinogenesis. Gene regulation by DNA methylation, histone acetylation, and noncoding RNAs upregulate or downregulate genes at the transcriptional or translational level. These changes do not affect the genetic sequence but lead to alterations in cellular function (36). CircRNAs have gained research attention among noncoding RNAs. The circRNA hsa_circ_0069244 inhibits the proliferation and migration of NSCLC cells by regulating XPC expression through miR-346 (37). Heterodimerization of circUGGT2 with KU70 and KU80, two key components of the NHEJ pathway, is associated with the progression of bladder cancer and resistance to cisplatin (38). Hsa_circ_0051488, derived from the gene ERCC1 associated with DDR, is downregulated in cells that undergo BPDE-induced malignant transformation. It exerts an anti-oncogenic effect through the miR-6717-5p–SATB2 axis (39). In this study, circ_0004470 was upregulated in several lung cancer–related samples and promoted malignant features such as BPDE-induced proliferation and migration in transformed 16HBE cells. These data suggest that circ_0004470 is involved in BPDE-induced lung carcinogenesis.

The epigenetic mechanisms regulating the response to genetic damage represent a novel avenue of investigation in the field of cancer research. The effects of genetic damage or epigenetic alterations are typically explored in isolation, as though there were an insurmountable barrier between genetics and epigenetics. In our preliminary investigations, we identified a correlation between the expression of miR-27a and the single nucleotide polymorphism (40). Recent research suggests that circRNAs regulate the DNA damage induced by pollutants. We found that circ_Cabin1 facilitates multi-organ DNA damage in PM2.5-exposed mice by suppressing the NHEJ pathway (41). Findings indicate that circ_0057504 enhances LIG4 expression, thereby inhibiting DNA damage in 16HBE cells exposed to carbon black nanoparticles (17). These studies indicate that circRNAs may be involved in the response to DNA damage induced by environmental carcinogens by targeting the DDR. Subsequently, we elucidated the role of circRNA regulation of DNA damage in lung carcinogenesis induced by the carcinogenic heavy metal cadmium (42). These studies provide evidence that circRNA regulation of DNA damage plays a significant role in lung carcinogenesis. However, further research is necessary to substantiate this hypothesis. The present study showed that circ_0004470 plays a role in the pathogenesis of lung cancer induced by BPDE *via* interactions with mRNAs or the XPC and DDB1 proteins. These interactions disrupt the normal DDR process, affecting DNA repair efficiency, cell cycle regulation, and the accumulation of DNA damage. These findings were validated in a diverse range of lung cancer-related cells, tissues, and animal models.

## Conclusions

In summary, we demonstrated that circ_0004470 regulates BPDE-induced DNA damage by interacting with XPC and DDB1, thereby facilitating the acquisition of cellular oncological phenotypes, including malignant proliferation, metastasis, and tumor-forming capacity. These processes ultimately lead to lung carcinogenesis. This study provides novel insight into the factors involved in lung carcinogenesis and a basis for the design of clinical treatment strategies.

## Experimental procedures

### Cell culture and BPDE treatment

Normal human bronchial epithelial 16HBE cells were cultured in modified Eagle's medium (MEM, 01-026-1ACS, Biological Industries), and human lung cancer cells were cultured in RPMI-1640 (Gibco) supplemented with 10% fetal bovine serum (FBS) and 1% penicillin-streptomycin (15140-122, Gibco) as described previously (43). BEAS-2B cells were cultured in bronchial epithelial cell medium (BEGM, #3211, ScienCell). Cells were passaged according to cell growth conditions, and third generation cells were used for functional experiments. The lung cancer cell lines A549, H446, and H1299 were obtained from the cell bank of the Institute for Chemical Carcinogenesis, Guangzhou Medical University. All cell lines were confirmed mycoplasma-negative through routine testing and used within 10 passages post-thaw.

BPDE (B1760, Sigma-Aldrich) was dissolved in dimethyl sulfoxide. Based on the actual exposure concentration of B[a]P in the population and the metabolic conversion rate of B[a]P to BPDE (10%), as well as the BPDE dose previously reported for inducing malignant transformation of human pulmonary bronchial epithelial cells (44, 45, 46, 47). BPDE exposure doses that did not have cytotoxic effects were determined using the CCK-8 and LDH assays. To establish a model of cellular malignant transformation, 16HBE cells were exposed to BPDE (0.00, 0.25, and 0.50 μM) for 40 generations. The optimal BPDE exposure concentration (0–1 μM, 24 h) and time (0–48 h, 1 μM) were selected for analysis of the dose or time effect relationship based on DNA damage assessed by the single cell gel electrophoresis assay and the expression of the γ-H2AX protein.

### Patient samples

A total of 18 lung cancer and adjacent tissue samples were collected from the Department of Thoracic Surgery of the Sixth Affiliated Hospital of Guangzhou Medical University. Normal tissue evaluation criteria were applied, ensuring that the samples were collected from areas >5 cm away from the tumor margin. Additionally, 14 lung tissue and blood samples were collected from normal healthy control patients. All lung cancer and adjacent tissue samples were stored in liquid nitrogen, whereas blood samples were stored at −80 °C. This study adhered to the principles of the Declaration of Helsinki and received approval from the Ethics Committee of Guangzhou Medical University (202101001). Prior to sampling, patient consent and approval were obtained.

### Cell viability and toxicity testing

Cell viability following BPDE treatment was evaluated using the CCK-8 assay (Dojindo), and cytotoxicity levels were determined using the LDH Assay Kit. In brief, cells were inoculated in triplicate in 96-well plates at a density of 1 × 10^4^ cells per well in a total volume of 100 μl MEM. After BPDE exposure, 10 μl of CCK8 reagent was added to each well and incubated at 37 °C for 2 h. For LDH, the cell supernatant was collected and centrifuged at 1000*g* for 15 min, and the supernatant was transferred to a new 96-well plate for the reaction according to the manufacturer's instructions. Finally, the absorbance of each group was measured at 450 nm using an enzyme marker (Bio Tek Epoch2).

### RNA extraction and qRT-PCR

The TRIzol reagent (Invitrogen) was used to isolate total RNA from cells and tissues. The cDNA was prepared from 1 μg of total RNA using the GoScript reverse transcription system (Promega) following the manufacturer's instructions. qRT-PCR was performed using GoTap qPCR Master Mix (Promega) in a QuantStudio5 real-time PCR system (Applied Biosystems). All primer sequences used in this study are shown in Table S1.

### Plasmid transfection and lentiviral infection

The siRNA sequence (Table S2) and the overexpression plasmid (pcDNA3.1(+)) for circ_0004470 were designed and constructed by Genepharma. Cells were transfected with Lipofectamine 3000 (Invitrogen) following the manufacturer's instructions. The circ_0004470 overexpression and interference lentiviral vectors were constructed by IGE. Puromycin was used to screen stably transfected cells. The transfection efficiency was determined by qRT-PCR.

### Western blot analysis

Cells or tissues were lysed on ice with RIPA lysis buffer (Beyotime Biotechnology), and after homogenization and centrifugation, total protein levels were quantified using the BCA Protein Assay kit (Thermo Fisher Scientific). After denaturation, proteins were separated by SDS-PAGE and transferred to a polyvinylidene difluoride membrane. The membrane was blocked in 1% bovine serum albumin for 1 h and incubated overnight with primary antibody. The following antibodies were used: γ-H2AX (1:1000, ab26350, Abcam), XPC (1; 1000, D1M5Y, Cell Signaling Technology), hHR23B (1:2000, ab86781, Abcam), DDB1 (1:1000, # 49708, SAB), CDT1 (1:1000, #3386, Cell Signaling Technology). The specificity of each antibody used was tested by comparing with the molecular weights of the ladder. The membranes were then incubated with goat anti-rabbit or goat anti-mouse IRDyeTM800CW secondary antibody at room temperature for 1 h. The membranes were scanned using an Odyssey infrared laser imaging system (LI-COR).

### Single-cell gel electrophoresis

The extent of DNA damage was determined using the single-cell gel electrophoresis assay. Briefly, single-cell suspensions were mixed with 0.5% low-melting-point agarose at 37 °C, transferred to precoated 1.0% agarose slides, immersed in lysing solution for 1 h, protected from light, and kept at 4 °C for 1 h. DNA was decondensed in precooled electrophoresis solution for 30 min and electrophoresed at 25 V for 30 min. After drying and staining, the DNA was examined and photographed under a fluorescence microscope (Eclipse Ci, Nikon).

### Cell cycle detection

For cell cycle testing, cells were collected and fixed overnight with 70% ethanol at 4 °C. After centrifugation, propidium iodide was added and incubated for 30 min at room temperature. Cell cycle detection was performed using a flow cytometer and results were analyzed using FlowJo 7.6.1 software (BD Biosciences).

### Plasmid treatment and host cell reactivation detection

The BPDE damage plasmid was prepared as described previously (48, 49, 50). The pCMVLuc reporter gene plasmid (IGE) was dissolved in TE buffer, and BPDE was added and incubated at 37 °C for 2 h to form the BPDE–DNA adduct. Plasmid DNA was precipitated with a 2-fold volume of ethanol, washed sufficiently, and then dissolved in in TE buffer for spare parts.

The DNA repair capacity of the cells was assessed using host cell reactivation and BPDE damage plasmids. Briefly, 4 μl (200 ng) of BPDE-modified pCMVluc plasmid was transfected into malignant transgenic cells with different treatments using the Lipofectamine 3000 reagent. Luciferase activity was measured, and total protein content was determined using the BCA assay. Activity was expressed as the number of relative luminescence units per μg protein.

### Immunofluorescence and fluorescence *in situ* hybridization

BPDE-exposed cells were fixed with 4% paraformaldehyde, permeabilized with 0.1% Triton X-100 for 10 min, blocked with 5% bovine serum albumin for 1 h, and incubated overnight at 4 °C in primary antibody followed by fluorescently labeled rabbit or mouse secondary antibody (567/488) for 2 h at 37 °C. For fluorescence *in situ* hybridization, the samples were hybridized overnight with a single circ_0004470-specific probe labeled with cyanine 3 (Cy3) at 4 °C. The cell nuclei were counterstained with DAPI. Images were captured using a Leica SP8 confocal microscope (Leica).

### Co-immunoprecipitation

Cells were transfected with a circRNA overexpression plasmid before exposure to BPDE. The cells were then lysed using the lysis reagent in the co-immunoprecipitation kit (BersinBio). Magnetic beads were crosslinked with XPC or hHR23B and IgG antibodies for 4 h at 4 °C. The antibody-coated beads and cell lysates were mixed overnight at 4 °C. The proteins bound to the specific antibodies were washed with buffer and subjected to Western blot analysis.

### Dual-luciferase reporter gene assay

The circ_0004470 dual luciferase vector containing the predicted XPC-binding site was constructed by IGE Biotechnology Co, Ltd and subsequently cotransfected with the aforementioned luciferase vector plasmid and the circRNA overexpression plasmid. Dual luciferase activity was assessed using the dual luciferase reporter assay system (Promega). GLuc luciferase activity was normalized to SEAP luciferase expression for each sample.

### RNA stability test

Cells were treated with AD (SBR00013, Sigma) to block transcription, and total RNA was collected after 0, 4, 8, and 12 h of culture. The effect of circ_0004470 overexpression on the stability of XPC or DDB1 mRNA was determined by qRT-PCR.

### RNA pulldown

The Pierce Magnetic RNA-Protein Pull-Down Kit (Thermo Fisher Scientific) was used to collect proteins bound to circ_0004470. The specific probe for circ_0004470 is shown in Table S3. Briefly, the biotin-labeled probe was incubated with magnetic beads to obtain probe–bead complexes. These complexes were then added to cell lysates and incubated for 2 h. Finally, the protein–probe–bead complexes were enriched using a magnetic rack, and the eluted protein products were used for mass spectrometry and Western blotting.

### RNA immunoprecipitation

RNA immunoprecipitation (RIP) assays were performed using the RIP Kit (BersinBio) following the manufacturer's instructions. Briefly, cell lysates were prepared in RIP lysis buffer, and after centrifugation at 4 °C, the supernatant was incubated with DDB1 antibody and control IgG at room temperature. The magnetic bead–antibody complexes were then washed and incubated with proteinase K. Finally, the immunoprecipitated RNA was purified and analyzed by qRT-PCR.

### Protein stability assessment

To determine the half-life of proteins, cells were transfected with a circ_0004470 overexpression plasmid and then treated with either the protein synthesis inhibitor cycloheximide (100 ng/ml, Sigma-Aldrich) or co-exposed to BPDE. Proteins were collected at 0, 6, 12, and 24 h. Comparative experiments were also conducted using the proteasome inhibitor MG-132 (50 μM, Sigma-Aldrich) or the lysosomal inhibitor chloroquine. Western blot analysis was performed on each group to assess changes in protein stability.

### Soft agar assay

The capacity of cells to grow in an anchorage-independent manner was assessed using the soft agar assay. Briefly, cells were resuspended in MEM containing 0.6% low melting point agarose and 10% FBS and then inoculated in 6-well plates precoated with MEM containing 1.2% low melting point agarose and 20% FBS. The plates were incubated under observation for 2 weeks, and colonies were manually counted under a microscope and photographed for documentation.

### Plate cloning assay

The colony forming ability of cells was assessed using a plate cloning assay. The cell suspension was diluted to a final concentration of 100 cells/ml and inoculated into a 6-well plate. The cells were observed for 1 to 2 weeks, fixed with 4% paraformaldehyde at room temperature, and stained with crystal violet solution for 30 min. The number of colonies was counted and recorded under the microscope.

### EdU assay

Cell proliferation was evaluated using the EdU kit (Ribobio). Cells exposed to different treatments were cultured in 96-well plates containing EdU-labeled medium. Cells were then treated with 4% paraformaldehyde and glycine, permeabilized with 0.5% Triton-X for 10 min, and incubated with Apollo staining solution for 30 min protected from light. Nuclei were labeled with Hoechst 33342 and finally photographed and documented under a fluorescence microscope (AMG EVOS).

### Transwell assay

Transwell chambers (Corning) were used for cell migration assays. Transwell chambers coated with Matrigel matrix gel (BD Biocoat) were used for cell invasion assays; serum-free MEM medium was added and hydrated at 37 °C for 1 h prior to the start of the experiments. In brief, 600 μl of MEM containing 20% serum was added to the lower chamber. Cell suspensions prepared with blank medium were inoculated into the upper chamber and cultured for 24 h. The cells in the upper chamber were then wiped off, and the cells that entered the lower chamber were fixed with paraformaldehyde (Beyotime) and stained with crystal violet for 15 min. Finally, five random areas were photographed under the microscope for analysis.

### Scratch experiment

The scratch assay was used to determine cell migration activity. Equal numbers of cells from each group were inoculated into 6-well plates. When the cells reached the bottom of the plate, a vertical line was drawn down the center of the wells with the tip of a 10 μl pipette and the culture was supplemented with serum-free medium. The initial scratch distance was recorded under a microscope, and the healing status of the cells in each group was further recorded after 24 h of continuous incubation.

### Tumor formation in nude mice

Cells from each group were resuspended in 0.1 ml PBS and injected into 5 week-old male BALB/c nude mice. Tumor volume (using the formula: Volume = (length × width^2^)/2) was measured every 3 days and body weight weekly. Mice were euthanized if weight loss exceeded 20% or tumor diameter surpassed 20 mm. Tumor weight was then measured. Data were analyzed statistically (mean ± SD by one-way ANOVA). A *p* value < 0.05 was considered significant. The experiment was approved by the Experimental Animal Ethics Committee of Guangzhou Medical University (GY2022-154).

### Exposure of A/J mice to B[a]P

For the short-term B[a]P repeated exposure experiments, female A/J mice aged 5 to 6 weeks were housed in the Animal Experiment Centre of Guangzhou Medical University (SPF grade). After 1 week of acclimatization, they were randomly divided into three groups: a control group treated with glyceryl trioctanoate and two experimental groups treated with B[a]P at doses of 0.2 mg/kg and 2 mg/kg. The mice were anesthetized with isoflurane gas and subjected to a nasal drip in the supine position every 3 days. Samples were collected on days 1, 3, 7, and 14 of the treatment.

For B[a]P chronic exposure experiments, A/J mice (female, 3–4 weeks of age) were transfected by nasal drip with control or overexpressed circ_0004470 AAV6 adenoviral vectors 3 weeks before B[a]P exposure. Mice received weekly nasal drops of 0.02 ml glyceryl trioctanoate at a B[a]P concentration of 0.2 mg/kg and sampled after 6 months of exposure.

Venous blood was collected from the posterior ocular plexus and stored at −80 °C. The mice were euthanized by spinal transection. The right lung was fixed in 5% neutral buffered formalin, dehydrated, degreased, embedded in paraffin, and stained with HE or used for immunohistochemistry. The left lung was collected and immediately frozen in liquid nitrogen. The Experimental Animal Ethics Committee of Guangzhou Medical University approved all animal experimental designs (GY2022-155,GY2022-156).

### Statistical analysis

Data were analyzed using GraphPad Prism 7.0 (GraphPad Software) and SPSS 23.0 (IBM) software. The results are presented as the mean ± SD. All experiments were performed in triplicate and repeated three times. Statistical analyses between two groups were conducted using Student's *t* test, and one-way ANOVA was used to determine statistical significance between more than two groups. *p*-values <0.05 were considered statistically significant.

## Data availability

All data needed to evaluate the conclusions of the study are presented in the paper and/or the Supplementary Materials.

## Supporting information

This article contains supporting information (seven supporting figures and four supporting table).

## Conflict of interest

The authors declare that they have no conflicts of interests with the contents of this article.
