# Supplementary material for: Circular RNA circ_0004470 accelerates the occurrence of lung cancer by promoting DNA damage and cell cycle arrest
Source: J Biol Chem. 2025 Mar 27;301(5):108456. doi: 10.1016/j.jbc.2025.108456 (PMC12147187; doi:10.1016/j.jbc.2025.108456)
Supplement: Supporting information [file mmc1.zip › Revised Supporting Information Figures.docx]

**Supplementary figures**

**Figure S1**

Figure S1. BPDE-transformed cells exhibit defects in the DNA damage response (related to Figure 1).

(**A**) CCK-8 and LDH assays were used to assess the effect of BPDE (0–2.00 μM) on the viability and toxicity of 16HBE and BEAS-2B cells after 24 h.

(**B**) Morphological alterations were observed in different generations of 16HBE cells chronically exposed to BPDE.

(**C**) The EdU assay was used to assess the proliferation of different generations of 16HBE cells chronically exposed to BPDE.

(**D**) The wound healing assay was used to detect the migratory capacity of transformed 16HBE cells treated with different concentrations of BPDE.

(**E**) Diagram of a BPDE-transformed nude mouse xenograft model.

(**F**) Western blot analysis of the expression of γ-H2AX in BEAS-2B cells treated with different concentrations of BPDE for 24 h.

(**G**) Immunofluorescence analysis of the expression of γ-H2AX in BEAS-2B cells treated with different concentrations of BPDE for 24 h.

(**H**) Single-cell gel electrophoresis assessment of DNA damage in BEAS-2B cells treated with different concentrations of BPDE for 24 h.

(**I**) Diagram of the short-term repeated B[a]P nasal drip exposure model in A/J mice.

(**J**) HE staining was used to assess the pathology of lung tissues from A/J mice exposed to different concentrations of B[a]P for different time periods.

Data represent mean ± SD from ≥3 biological replicates. Statistical significance was determined by Student’s t-test (two groups) or one-way ANOVA (≥3 groups): **p* < 0.05, ***p* < 0.01, ****p* < 0.001.

**Figure S2**

**Figure S2. Verification of the cyclic characteristics of circ_0004470 (related to Figure 2 ).**

**(A** and **B)**  Heatmap (A) and scatterplot (B) analysis of differentially expressed circRNAs in 16HBE cells treated with or without BPDE.

(**C**) Actinomycin D treatment (left) or the RNase R exonuclease resistance assay (right) were used to assess the stability of circ_0004470 and its parent gene MYH9 mRNA.

**(D** and **E**) Fluorescence in situ hybridization (D) or nucleoplasmic separation combined with qRT-PCR (E) were used to determine the subcellular localization of circ_0004470.

(**F**) qRT-PCR was used to detect the expression of circ_0004470 in BEAS-2B cells exposed to BPDE at different concentrations (0–1μM, 24 h) and times (0–48 h, 1 μM).

(**G**) Lentiviral transfection was used to construct stable circ_0004470 overexpression (lv-oe) and knockdown (sh-si) cell lines.

Data represent mean ± SD from ≥3 biological replicates. Statistical significance was determined by Student’s t-test (two groups) or one-way ANOVA (≥3 groups): **p* < 0.05, ***p* < 0.01, ****p* < 0.001.

**Figure S3**

**Figure S3. Construction of a circ_0004470 knockdown or overexpression system (related to Figure 3).**

**(A)** Efficiency of transient knockdown or overexpression of circ_0004470 in 16HBE and BEAS-2B cells.

**(B)**  Circ_0004470 expression in knockdown or overexpression systems of circ_0004470 in BPDE-exposed 16HBE or BEAS-2B cells.

**(C)** Immunofluorescence analysis of the effect of circ_0004470 on γ-H2AX expression in BPDE-treated BEAS-2B cells.

**(D)** Single cell gel electrophoresis assay of the effect of circ_0004470 on DNA damage in BEAS-2B cells upon acute exposure to BPDE.

**(E**) Immunofluorescence analysis of the effect of circ_0004470 on γ-H2AX expression in BPDE malignantly transformed cells.

Data represent mean ± SD from ≥3 biological replicates. Statistical significance was determined by Student’s t-test (two groups) or one-way ANOVA (≥3 groups): ***p* < 0.01, ****p* < 0.001.

**Figure S4**

**Figure** **S4. Decreased expression of XPC during the malignant transformation of human lung bronchial epithelial cells (related to Figure 4).**

1. Expression of XPC mRNA in different generations of 16HBE cells chronically exposed to BPDE.

**(B)** Western blot analysis of XPC expression in different generations of 16HBE cells chronically exposed to BPDE.

**(C)** Lentiviral vector constructs for stable overexpression of XPC (left) were constructed and their XPC overexpression efficiency was analyzed (right).

**(D** and **E)** Immunofluorescence staining (D) and host cell reactivation assays (E) were used to evaluate the effect of XPC on DNA damage and repair capability in 16HBE cells.

**(F)** Western blot analysis of the effect of circ_0004470 knockdown combined with BPDE exposure on XPC expression in 16HBE cells.

Data represent mean ± SD from ≥3 biological replicates. Statistical significance was determined by Student’s t-test (two groups) or one-way ANOVA (≥3 groups): ***p* < 0.01, ****p* < 0.001.

**Figure S5**

**Figure S5. The carcinogenic effect of circ_0004470 is associated with the inhibition of XPC function (related to Figure 5).**

**(A)** Kaplan–Meier survival analysis of the effect of XPC expression levels in lung cancer cases based on The Cancer Genome Atlas (TCGA) database.

**(B)** EdU assays was used to assess the effect of circ_0004470 co-transfection with XPC on the malignant proliferative capacity of BPDE-transformed cells.

(**C** and **D**) Soft agar clone formation (C) and plate cloning (D) assays were used to evaluate the effect of co-transfection of circ_0004470 with XPC on the proliferation of A549 and H1299 cells.

(**E** to **G)** Transwell (E and F) and wound healing (G) assays were used to assess the effect of co-transfection of circ_0004470 with XPC on the metastatic ability of A549 and H1299 cells.

Data represent mean ± SD from ≥3 biological replicates. Statistical significance was determined by Student’s t-test (two groups) or one-way ANOVA (≥3 groups): ***p* < 0.01, ****p* < 0.001.

**Figure S6**

**Figure S6. DDB1 affects the expression of CDT1, a cell cycle regulator involved in the DNA damage response (related to Figure 6).**

(**A**) Schematic of RNA pull-down and mass spectrometry analysis of circ_0004470 (left) and verification of binding proteins using the silver staining assay (right).

(**B**) The RIP assay was used to validate the interaction between DDB1 and circ_0004470.

(**C**) Western blot analysis of DDB1 expression in different generations of 16HBE cells chronically exposed to BPDE.

(**D**) Construction and validation of DDB1 knockdown and overexpression systems.

(**E**) Single-cell gel electrophoresis (left) and immunofluorescence staining (right) were used to assess the effect of DDB1 knockdown on BPDE-induced DNA damage in 16HBE cells.

(**F**) Flow cytometry analysis of the effect of DDB1 knockdown on the cell cycle of 16HBE cells exposed to BPDE.

(**G**) Kaplan–Meier survival analysis of CDT1 expression in lung cancer cases based on TCGA database.

(**H**) Western blot analysis of the effect of DDB1 knockdown on CDT1 protein expression in BPDE-exposed 16HBE cells.

(**I**) Western blot analysis of the effect of knockdown of circ_0004470 combined with BPDE exposure on DDB1 protein expression. Data represent mean ± SD from ≥3 biological replicates. Statistical significance was determined by Student’s t-test (two groups) or one-way ANOVA (≥3 groups): **p* < 0.05, ***p* < 0.01, ****p* < 0.001.

**Figure S7**

**Figure S7. Circ _ 0004470 accelerates lung cancer progression by inhibiting DDB1 function (related to Figure 7).**

(**A**) EdU assays were used to assess the effect of co-transfection of circ_0004470 and DDB1 on the malignant proliferative capacity of BPDE-transformed cells.

(**B**) Wound healing assays were used to assess the effect of co-transfection of circ_0004470 and DDB1 on the migration capacity of BPDE-transformed cells.

(**C**) Flow cytometry was used to examine the effect of co-transfection of circ_0004470 and DDB1 on the cell cycle of A549 and H1299 cells.

(**D** and **E**) Soft agar clone formation (D) and plate cloning (F) assays were used to evaluate the effect of co-transfection of circ_0004470 with DDB1 on the proliferation of A549 and H1299 cells.

(**F** to **H**) Transwell (F and G) and wound healing (H) assays were used to assess the effect of co-transfection of circ_0004470 with XPC on the metastatic ability of A549 and H1299 cells.

Data represent mean ± SD from ≥3 biological replicates. Statistical significance was determined by Student’s t-test (two groups) or one-way ANOVA (≥3 groups): **p* < 0.05, ***p* < 0.01, ****p* < 0.001.
