# Supplementary material for: Circular RNA circ_0004470 accelerates the occurrence of lung cancer by promoting DNA damage and cell cycle arrest
Source: J Biol Chem. 2025 Mar 27;301(5):108456. doi: 10.1016/j.jbc.2025.108456 (PMC12147187; doi:10.1016/j.jbc.2025.108456)
Supplement: Supporting information [file mmc1.zip › Revised Supporting Information Table.docx]

**Supplementary Tables**

**Table S1. Primer sequences for quantitative real-time PCR**

| **Gene** | **Species** | **Forward primer** | **Reverse primer** |
| --- | --- | --- | --- |
| Circ0004470 | Human | TATGCCATCACAGACACCGC | AAGCCACTCTTGTCGGAAGG |
| MYH9 | Human | GTATGGGTGCCTTCCGACAA | GCGGTGTCTGTGATGGCATA |
| U6 | Human | CTCGCTTCGGCAGCACA | AACGCTTCACGAATTTGCGT |
| GAPDH | Human | GCAAGTCAGCCGCATCTTCT | GCGCCCAATCACGACCAAATAC |
| XPC | Human | GACCTCAAGAAGGCACACCA | TCTTTTCACTTCAACCTCTTCCCA |
| CDT1 | Human | AGGTGCTGGCGGAGATGTTC | TGGCAAAGGTGGGCGTCTC |
| DDB1 | Human | GAAGACCAAGGGCGACTTCA | ACAGCACTCATCCAGTTGGG |
| circ0004470 | Mouse | GGCAAGAAGAGGCACGAGAT | TCAAAGCCATTCTTGCTGGA |
| Xpc | Mouse | ACCACCTCCTCCACCTTGACTG | TCTGGCTGCTTCTGCTTGTGAAC |
| Cdt1 | Mouse | GTCAAGGAACAGCACAAGGTCTTC | TCAGGCACCTCGTCCACATTG |
| Ddb1 | Mouse | TGTTGAGCGACCGTAAGAAGATGAC | GGACTTCAATGCGGCTGGAGAG |

**Table S2. Sequences of siRNAs**

| **Gene** | **siRNA Sequence** |
| --- | --- |
| circ0004470-siRNA 1 | GGAGUAUGAUGCAAGGUCCTT |
| circ0004470-siRNA 2 | AAUAAGCCCUGCCCAAAGATT |
| DDB1 siRNA 1 | GAGGUGCACAACCUACUUAdTdT |
| DDB1 siRNA 2 | GCGAGAGCAUUGACAUCAUdTdT |
| DDB1 siRNA 3 | GCUCAACGUUGACAGUAAUdTdT |

**Table S3. Sequences of RNA pull-down probes**

| **Gene symbol** | **Label** | **Probe sequence** |
| --- | --- | --- |
| Circ_0004470 | 5`Biotin | TGCCATGGTGACTTATAGCCAGGACCTTGCATCATACTCCTGTAGGCGGT |

**Table S4. Information on circMYH9 (hsa_circ_0004470) from circBank**

| **circBank ID** | hsa_circMYH9_006 |
| --- | --- |
| **circBase ID** | hsa_circ_0004470 |
| **Position** | chr22: 36737414-36745300 strand: - |
| **Length** | 509 |
| **Host gene Symbol** | MYH9 |
| **bestTranscript** | NM_002473 |
| **Annotation** | ANNOTATED, CDS, coding, INTERNAL, OVCODE, OVEXON, UTR5 |
| **RNA sequences** | GTCCTGGCTATAAGTCACCATGGCACAGCAAGCTGCCGATAAGTATCTCTATGTGGATAAAAACTTCATCAACAATCCGCTGGCCCAGGCCGACTGGGCTGCCAAGAAGCTGGTATGGGTGCCTTCCGACAAGAGTGGCTTTGAGCCAGCCAGCCTCAAGGAGGAGGTGGGCGAAGAGGCCATCGTGGAGCTGGTGGAGAATGGGAAGAAGGTGAAGGTGAACAAGGATGACATCCAGAAGATGAACCCGCCCAAGTTCTCCAAGGTGGAGGACATGGCAGAGCTCACGTGCCTCAACGAAGCCTCGGTGCTGCACAACCTCAAGGAGCGTTACTACTCAGGGCTCATCTACACCTATTCAGGCCTGTTCTGTGTGGTCATCAATCCTTACAAGAACCTGCCCATCTACTCTGAAGAGATTGTGGAAATGTACAAGGGCAAGAAGAGGCACGAGATGCCCCCTCACATCTATGCCATCACAGACACCGCCTACAGGAGTATGATGCAAG |
| **conserved mm9**  **circRNA** | >chr15_77807867_77813231_-  GTCCTGGCCGCAAGTCACCATGGCTCAGCAGGCTGCAGACAAGTACCTCTATGTGGATAAAAACTTCATCAATAACCCGCTGGCCCAAGCTGACTGGGCTGCCAAGAAGTTGGTATGGGTGCCTTCCAGCAAGAATGGCTTTGAACCAGCTAGCCTCAAGGAGGAGGTGGGAGAAGAGGCCATTGTAGAGCTGGTAGAGAATGGGAAGAAGGTGAAGGTGAACAAGGACGACATCCAGAAGATGAACCCACCCAAGTTCTCCAAGGTGGAGGACATGGCAGAGCTCACGTGCCTCAACGAAGCTTCGGTGCTGCACAACCTCAAGGAGCGATACTACTCAGGGCTTATCTACACCTATTCAGGCCTGTTCTGTGTGGTCATCAACCCTTATAAGAACCTGCCCATCTACTCAGAGGAGATCGTGGAGATGTACAAGGGCAAGAAGAGGCACGAGATGCCACCCCACATCTACGCCATCACAGATACTGCCTACCGGAGCATGATGCAGG |
